# Enhanced alcohol metabolism and sleep quality with continuous positive airway pressure following alcohol consumption

**DOI:** 10.1038/s41598-025-98702-9

**Published:** 2025-04-28

**Authors:** Hyun Jun Kim, Do-Yang Park, Wee Gyo Lee, Kang Il Lee, Jin Ji Jung, Han Sang Lee, Sang In Hwang, Ji Hyun Park, Bumhee Park

**Affiliations:** 1 Sleep Center, Ajou University Hospital, 164 World cup-ro, Yeongtong-gu, Suwon-si, Gyonggi-do 16499 Republic of Korea; 2 Department of Otorhinolaryngology - Head and Neck Surgery, Ajou University School of Medicine, Suwon, Republic of Korea; 3 Department of Laboratory Medicine, Ajou University School of Medicine, Suwon, Republic of Korea; 4 Office of Biostatistics, Medical Research Collaborating Center, Ajou Research Institute for Innovative Medicine, Ajou University Medical Center, Suwon, Republic of Korea; 5 Department of Biomedical Informatics, Ajou University School of Medicine, Suwon, Republic of Korea

**Keywords:** Acetaldehyde, Ethanol, Hypoxia, Oxygen, Translational research, Hepatology

## Abstract

We aimed to examine the effect of continuous positive airway pressure on sleep quality and alcohol metabolism after alcohol consumption. Men (n = 53) aged ≥ 19 years with sleep disorders who regularly consumed an average of ≥ 1.0 g of alcohol/kg of bodyweight, were free of serious diseases (including liver disorders), and underwent polysomnography and continuous positive airway pressure titration between January 2016 and July 2021 were included. Participants drank a high dose of a traditional Korean liquor at a rate of 1.0 g/kg of bodyweight for 1 h. The main outcome measures included polysomnography results and blood and breath ethanol and acetaldehyde concentrations after alcohol consumption before and after sleep. Statistical analyses were performed using R software, version 4.0.5 (R Foundation, Vienna, Austria). Continuous positive airway pressure enhanced sleep quality after alcohol consumption, with oxygen significantly improving the metabolism of acetaldehyde over that of ethanol. Breath and blood sample analyses and polysomnography results revealed that continuous positive airway pressure improved sleep quality by reducing apnea–hypopnea index by 27.32 ± 24.87 (*p* < 0.001), increasing rapid eye movement sleep by 2.08 ± 6.74% (*p* < 0.05), and enhancing acetaldehyde breakdown by 21.2% (*p* < 0.001), while its effect on ethanol breakdown (4–5%) was not statistically significant. Continuous positive airway pressure is recommended after alcohol consumption for individuals with sleep apnea to enhance sleep quality.

**Supplementary Information:**

The online version contains supplementary material available at 10.1038/s41598-025-98702-9.

Alcohol produces various metabolic byproducts during its breakdown, causing short-term effects such as hangovers and increasing the risk of long-term health problems, including cancer, cardiovascular diseases, and liver disease^1–3^. It also negatively impacts sleep quality^4–6^. While alcohol initially induces sleep, it ultimately worsens sleep apnea and overall sleep quality. A meta-analysis of 13 studies found that alcohol increases the apnea–hypopnea index (AHI) by 3.98 and reduces the lowest oxygen saturation by 2.72%. Additionally, alcohol delays sleep onset, reduces sleep efficiency and REM sleep, and increases arousals and REM latency, with REM sleep being most affected at moderate and high doses^6^. Notably, alcohol’s impact on sleep varies throughout the night, promoting sleep in the first half but causing arousal and poorer sleep quality in the second half.

Therefore, to mitigate these adverse effects, expeditious alcohol metabolism in the liver is crucial, and oxygen is known to facilitate it^7–9^. Intermittent hypoxia caused by sleep apnea may inhibit the breakdown of alcohol metabolites, exacerbating detrimental effects.

The adverse effects of alcohol consumption on sleep quality and the beneficial impact of continuous positive airway pressure (CPAP) in the treatment of sleep apnea are widely acknowledged. However, the impact of CPAP on sleep apnea after alcohol consumption on sleep quality and alcohol metabolism is unknown. To address this gap in the literature, we aimed to examine the impact of CPAP on sleep quality after alcohol consumption in high doses and evaluate potential alterations in alcohol breakdown and metabolism. To this end, we conducted sleep quality assessments in patients with and without CPAP and with and without alcohol consumption.

## Results

The mean age of the participants was 39.53 ± 8.05 years, and the mean apnea–hypopnea index (AHI) was 31.39 ± 28.17 (Table 1).

**Table 1:** Basic characteristics of the participants.

Characteristic	
N	53
Age (years)	39.53 ± 8.05
Bodyweight (kg)	81.07 ± 11.80
Height (m)	1.74 ± 0.05
BMI (kg/m^2^)	26.59 ± 3.40
Neck circumference (cm)	38.51 ± 2.65
Waist circumference (cm)	93.96 ± 9.60
Hip circumference (cm)	99.38 ± 11.10
AHI (events/h)	31.39 ± 28.17
RDI (events/h)	37.49 ± 26.51
Mean oxygen saturation (%)	94.77 ± 2.44
Lowest oxygen saturation (%)	82.23 ± 8.81
Relative snore time (%)	37.56 ± 20.20

AHI, apnea–hypopnea index; BMI, body mass index; RDI, respiratory disturbance index.

All measurements are reported as means ± standard deviations.

### Analysis of alcohol metabolism

The blood alcohol level (BAL), breath alcohol concentration (BrAC), and acetaldehyde in the blood (AcAld) were lower in the morning (after sleeping) than at night (before sleeping) in both the polysomnography with alcohol (PSG_alc_) and CPAP titration with alcohol (CPAP_alc_) groups. Specifically, AcAld was lower in the CPAP_alc_ group (29.33%) than in the PSG_alc_ group (50.53%), indicating that CPAP enhanced alcohol metabolism, especially acetaldehyde metabolism (Table 2).

**Table 2:** Ethanol and acetaldehyde concentrations.

		PSG_alc_		CPAP_alc_	
		Night	Morning	Night	Morning
BrAC	Mean ± SD (g%)	0.099 ± 0.029	0.010 ± 0.016	0.087 ± 0.034	0.005 ± 0.010
	%	100	8.94	100	4.73
BAL	Mean ± SD (mg/dL)	102.72 ± 31.68	15.19 ± 16.88	94.48 ± 35.22	10.17 ± 13.36
	%	100	14.34	100	9.59
AcAld	Mean ± SD (mg/L)	5.56 ± 2.29	2.53 ± 1.18	5.21 ± 2.04	1.50 ± 1.15
	%	100	50.53	100	29.33

BrAC, breath alcohol concentration; BAL, blood alcohol level; AcAld, acetaldehyde concentration in the blood; PSG_alc_, polysomnography after alcohol consumption; CPAP_alc_, continuous positive airway pressure titration after alcohol consumption; SD, standard deviation.

Additionally_,_ significant differences were found in the BrAC, BAL, and AcAld when two measurements obtained at night and in the morning were compared between the PSG_alc_ and CPAP_alc_ groups (Supplementary Table 1). Discrepancies among the BrAC, BAL, and AcAld values measured at night accounted for approximately 29–34% of the measurements in the PSG_alc_ group (Supplementary Table 1). As the BrAC and BAL measurements obtained in the morning were zero, it was not possible to perform a relative change analysis. No significant differences were detected in the aspartate transaminase (AST), alanine transaminase (ALT), and r-glutamyl transferase (r-GT) values between the nighttime and morning measurements, regardless of CPAP use (Supplementary Table 2).

**Fig. 1:**
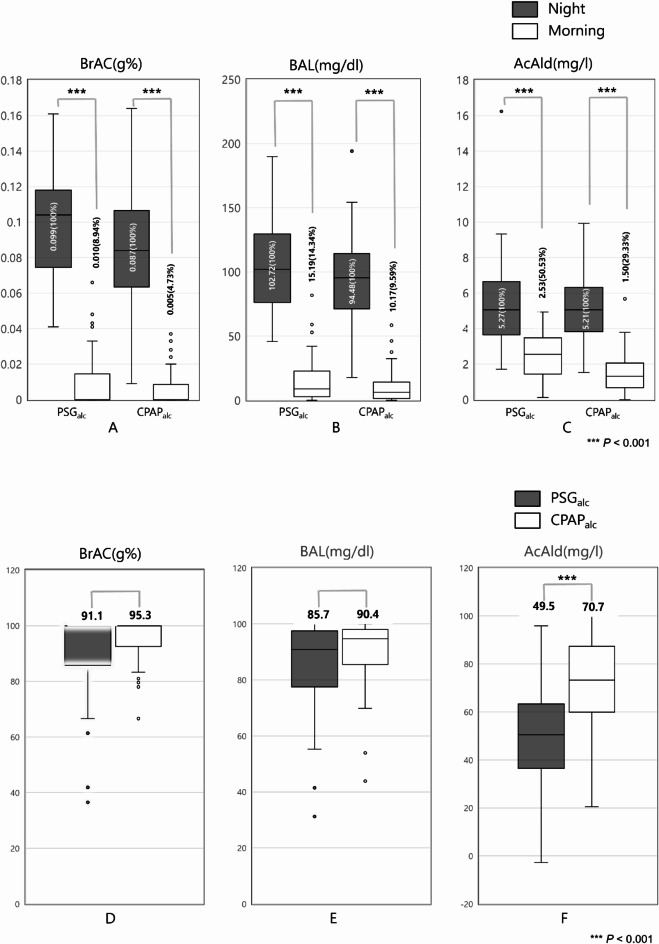
Changes in ethanol and acetaldehyde concentrations. (**A**–**C**) Breath alcohol concentration (BrAC) (**A**), blood alcohol level (BAL) (**B**), and acetaldehyde concentration in the blood (AcAld) (**C**) after alcohol consumption, measured at night and in the morning on two consecutive days, with and without CPAP. (**D**–**F**) Percentage of decrease in BrAC (**D**), BAL (**E**), and AcAld (**F**) from night to morning in the PSG_alc_ and CPAP_alc_ groups. *p < 0.05. CPAP_alc_, continuous positive airway pressure titration after alcohol consumption; CPAP_ctr_, continuous positive airway pressure titration without alcohol consumption; PSG_alc_, polysomnography after alcohol consumption; PSG_ctr_, polysomnography without alcohol consumption.

Figure 1A–C illustrate the results of the BrAC, BAL, and AcAld measurements at night and in the morning after alcohol consumption in the PSG_alc_ and CPAP_alc_ groups. Changes in values were calculated relative to the nighttime measurements. Irrespective of CPAP usage, all parameter values exhibited a substantial decrease in the morning compared with those at night. The BrAC was decreased to 8.94% and 4.73% in the PSG_alc_ and CPAP_alc_ groups, respectively (Fig. 1A). Similarly, the BAL decreased to 14.34% and 9.59%, respectively (Fig. 1B), and the AcAld decreased to 50.53% and 29.33%, respectively (Fig. 1C).

Figure 1D–F show the differences between the PSG_alc_ and CPAP_alc_ groups in the changes from the nighttime to morning measurements_._ BrAC was decreased by 91.1% and 95.3% in the PSG_alc_ and CPAP_alc_ groups, respectively, indicating a 4.2% additional reduction due to CPAP use (Fig. 1D). However, this difference was not statistically significant. Similarly, the BAL decreased by 85.7% and 90.4%, respectively, suggesting a 4.7% additional reduction (Fig. 1E), though this difference was not statistically significant. Finally, the AcAld decreased by 49.5% and 70.7%, respectively, indicating a 21.2% additional reduction. These findings indicate that CPAP is effective in reducing BrAC, BAL, and especially AcAld (Fig. 1F).

### Analysis of sleep quality

We compared the sleep quality among the four groups. Sleep quality was determined considering wake after sleep onset (WASO), total sleep time (TST), sleep onset latency (SOL), sleep efficiency (SE), the arousal index, latency to rapid eye movement (REM), AHI, respiratory disturbance index (RDI), supine AHI, snoring rate, mean oxygen saturation, lowest oxygen saturation, and the proportion of time spent in the W, N1, N2, N3, and R sleep stages.

The results revealed no significant differences in the AHI or RDI between the PSG_ctr_ and PSG_alc_ groups, suggesting that alcohol consumption does not affect sleep apnea severity. Additionally, the overall increase in AHI due to alcohol was 1.30, while REM AHI increased by 3.01, both of which were not statistically significant. However, the PSG_alc_ group showed a 7.91 ± 18.22% increase in the snoring rate, as well as a decrease of 1.49 ± 1.59% and 4.77 ± 5.11% in the mean and lowest oxygen saturation, respectively. Additionally, REM latency was increased by 25.84 ± 64.01 min, REM duration was decreased by 1.76 ± 6.31%, and N3 was increased by 1.98 ± 5.61. This implies that alcohol increases N3 and suppresses REM sleep (Supplementary Table 3).

In the CPAP_ctr_ group, the AHI was significantly decreased to a normal level (*M* = 4.60) compared with that in the PSG_ctr_ group (*M* = 31.39). Additionally, the durations of N2, N3, and REM were increased, whereas those of N1, as well as the arousal index, decreased (Supplementary Table 4).

The CPAP_alc_ group showed a lower AHI (*M* = 5.37) than the PSG control (PSG_ctr_) group (*M* = 31.39) did, indicating that the AHI in the CPAP_alc_ group was close to normal. The CPAP_alc_ group had higher N2 and N3 durations and lower N1 durations and arousal index than did the PSG_ctr_ group (Table 3).

**Table 3:** Changes in sleep status between the PSG_ctr_ and CPAP_alc_ groups.

Patient characteristic	PSG_ctr_	CPAP_alc_	PSG_ctr__−_ CPAP_alc_	95% confidence interval		*p* value
	Mean ± SD	Mean ± SD	Mean ± SD	L	U	
TST, min	369.98 ± 45.40	371.67 ± 31.67	-1.69 ± 50.57	-15.63	12.24	0.808
Sleep efficiency, %	87.97 ± 10.81	88.65 ± 7.34	-0.68 ± 12.03	-3.99	2.64	0.684
N1, %	19.93 ± 12.48	10.87 ± 4.06	9.15 ± 12.49	5.70	12.59	0.000***
N2, %	46.47 ± 13.38	51.95 ± 8.84	-5.48 ± 15.51	-9.75	-1.20	0.013*
N3, %	4.62 ± 5.23	8.55 ± 6.43	-3.93 ± 5.18	-5.36	-2.51	0.000***
REM, %	18.61 ± 6.40	18.93 ± 5.81	-0.31 ± 8.46	-2.64	2.02	0.789
W, %	10.41 ± 10.47	9.80 ± 6.29	0.62 ± 12.23	-2.76	3.99	0.716
WASO, min	42.66 ± 41.40	41.02 ± 24.17	1.64 ± 48.68	-11.78	15.06	0.807
SOL, min	9.63 ± 16.47	6.59 ± 9.06	3.04 ± 11.49	-0.13	6.21	0.060
REM latency, min	99.19 ± 54.62	113.76 ± 53.26	-14.58 ± 75.19	-35.30	6.15	0.164
AHI, /h	31.39 ± 28.17	5.37 ± 4.92	26.02 ± 25.23	19.07	32.98	0.000***
Supine AHI, /h	41.21 ± 35.87	5.95 ± 5.54	35.26 ± 32.74	26.24	44.29	0.000***
RDI, /h	37.49 ± 26.51	7.27 ± 5.14	30.22 ± 23.36	23.78	36.66	0.000***
Arousal index, /h	34.56 ± 22.19	15.79 ± 5.86	18.77 ± 20.89	13.01	24.53	0.000***
Mean oxygen saturation, %	94.77 ± 2.44	95.88 ± 1.22	-1.11 ± 2.05	-1.67	-0.54	0.000***
Lowest oxygen saturation, %	82.23 ± 8.81	86.47 ± 7.00	-4.25 ± 7.21	-6.23	-2.26	0.000***

All measurements are reported as means ± standard deviations. * *p* < 0.05, *** *p* < 0.001.

AHI, apnea–hypopnea index; PSG_ctr_, polysomnography without alcohol consumption; CPAP_alc_, continuous positive airway pressure titration after alcohol consumption; N1, non-rapid eye movement sleep, stage 1; N2, non-rapid eye movement sleep, stage 2; N3, non-rapid eye movement sleep, stage 3; REM, rapid eye movement; SOL, sleep onset latency; RDI, respiratory disturbance index; SD, standard deviation; TST, total sleep time; WASO, wake after sleep onset.

The CPAP_alc_ group showed a lower AHI (*M* = 5.37) than did the PSG_alc_ group (*M* = 32.69). Additionally, N2, N3, and REM durations were higher in the CPAP_alc_ group than in the PSG_alc_ group (Table 4).

**Table 4:** Changes in sleep status between the PSG_alc_ and CPAP_alc_ groups.

Patient characteristic	PSG_alc_	CPAP_alc_	PSG_alc__−_ CPAP_alc_	95% confidence interval		*p* value
	Mean ± SD	Mean ± SD	Mean ± SD	L	U	
TST, min	372.93 ± 32.89	371.67 ± 31.67	1.26 ± 21.91	-4.78	7.30	0.678
Sleep efficiency, %	88.91 ± 7.88	88.65 ± 7.34	0.26 ± 5.50	-1.25	1.78	0.730
N1, %	20.43 ± 12.47	10.78 ± 4.06	9.64 ± 13.17	6.01	13.27	0.000***
N2, %	47.61 ± 10.55	51.95 ± 8.84	-4.34 ± 11.91	-7.63	-1.06	0.010**
N3, %	6.29 ± 6.23	8.55 ± 6.43	-2.26 ± 6.02	-3.92	-0.60	0.008
REM, %	16.85 ± 6.78	18.93 ± 5.81	-2.08 ± 6.74	-3.93	-0.22	0.029*
W, %	8.83 ± 6.27	9.80 ± 6.29	-0.97 ± 5.37	-2.45	0.51	0.196
WASO, min	34.89 ± 22.42	41.02 ± 24.17	-6.13 ± 22.54	-12.34	0.08	0.053
SOL, min	11.10 ± 16.58	6.59 ± 9.06	4.51 ± 10.53	1.60	7.41	0.003
REM latency, min	125.03 ± 54.78	113.76 ± 53.26	11.26 ± 71.26	-8.38	30.91	0.255
AHI, /h	32.69 ± 27.95	5.37 ± 4.92	27.32 ± 24.87	20.47	34.18	0.000***
Supine AHI, /h	41.22 ± 32.64	5.95 ± 5.54	35.27 ± 29.16	27.23	43.30	0.000***
RDI, /h	39.11 ± 25.75	7.27 ± 5.14	31.84 ± 22.69	25.59	38.09	0.000***
Arousal index, /h	33.93 ± 20.50	15.79 ± 5.86	18.14 ± 19.78	12.69	23.60	0.000***
Mean oxygen saturation, %	93.29 ± 3.46	95.88 ± 1.22	-2.60 ± 3.00	-3.42	-1.77	0.000***
Lowest oxygen saturation, %	77.45 ± 10.63	86.47 ± 7.00	-9.02 ± 8.71	-11.42	-6.62	0.000***

All measurements are reported as means ± standard deviations. * *p* < 0.05, ** *p* < 0.01, *** *p* < 0.001.

AHI, apnea–hypopnea index, PSG_alc_, polysomnography after alcohol consumption, CPAP_alc_, continuous positive airway pressure titration after alcohol consumption, N1, non-rapid eye movement sleep, stage 1; N2, non-rapid eye movement sleep, stage 2; N3, non-rapid eye movement sleep, stage 3; PSG_alc_, polysomnography after alcohol consumption; REM, rapid eye movement; SOL, sleep onset latency; RDI, respiratory disturbance index; SD, standard deviation; TST, total sleep time; WASO, wake after sleep onset.

No significant differences were observed in the AHI, RDI, or N2 duration between the CPAP control (CPAP_ctr_) and CPAP_alc_ groups. However, we found significant differences between these groups regarding oxygen saturation, N3, and REM durations. For oxygen saturation, both the mean and lowest values were lower in the CPAP_alc_ group than in the CPAP_ctr_ group. Moreover, the CPAP_alc_ group revealed a higher N3 and REM latency but a lower REM duration than did the CPAP_ctr_ group (Table 5).

**Table 5:** Changes in sleep status between the CPAP_ctr_ and CPAP_alc_ groups.

Patient characteristic	CPAP_ctr_	CPAP_alc_	CPAP_ctr_ − CPAP_alc_	95% confidence interval		*p* value
	Mean ± SD	Mean ± SD	Mean ± SD	L	U	
TST, min	376.10 ± 29.75	371.67 ± 31.67	4.42 ± 26.78	-2.96	11.81	0.235
Sleep efficiency, %	89.60 ± 7.09	88.65 ± 7.34	0.95 ± 6.35	-0.80	2.70	0.281
N1, %	10.64 ± 4.21	10.78 ± 4.06	-0.15 ± 4.18	-1.30	1.01	0.801
N2, %	51.11 ± 8.40	51.95 ± 8.84	-0.84 ± 10.32	-3.68	2.01	0.558
N3, %	6.52 ± 5.63	8.55 ± 6.43	-2.03 ± 6.27	-3.76	-0.30	0.022*
REM, %	21.97 ± 6.01	18.93 ± 5.81	3.04 ± 6.67	1.20	4.88	0.002**
W, %	9.76 ± 6.28	9.80 ± 6.29	-0.04 ± 6.96	-1.95	1.88	0.969
WASO, min	41.08 ± 26.58	41.02 ± 24.17	0.06 ± 27.67	-7.57	7.69	0.988
SOL, min	6.86 ± 12.28	6.59 ± 9.06	0.27 ± 6.81	-1.61	2.15	0.774
REM latency, min	81.91 ± 33.96	113.76 ± 53.26	-31.86 ± 55.64	-47.19	-16.52	0.000***
AHI, /h	4.60 ± 4.59	5.37 ± 4.92	-0.76 ± 3.38	-1.69	0.17	0.106
Supine AHI, /h	5.13 ± 5.40	5.95 ± 5.54	-0.82 ± 5.10	-2.23	0.58	0.245
RDI, /h	7.10 ± 6.13	7.27 ± 5.14	-0.17 ± 4.01	-1.28	0.93	0.754
Arousal index, /h	17.04 ± 7.05	15.79 ± 5.86	1.25 ± 6.46	-0.53	3.03	0.163
Mean oxygen saturation, %	96.52 ± 0.91	95.88 ± 1.22	0.64 ± 0.86	0.40	0.87	0.000***
Lowest oxygen saturation, %	89.17 ± 5.04	86.47 ± 7.00	2.70 ± 5.72	1.12	4.27	0.001**
Optimal pressure, cmH_2_O	8.24 ± 2.48	8.65 ± 2.61	-0.41 ± 1.39	-0.80	-0.03	0.035*

All measurements are reported as means ± standard deviations. * *p* < 0.05, ** *p* < 0.01, *** *p* < 0.001.

AHI, apnea–hypopnea index; CPAP_ctr_, continuous positive airway pressure titration without alcohol consumption; CPAP_alc_, continuous positive airway pressure titration after alcohol consumption; N1, non-rapid eye movement sleep, stage 1; N2, non-rapid eye movement sleep, stage 2; N3, non-rapid eye movement sleep, stage 3; REM, rapid eye movement; RDI, respiratory disturbance index; SD, standard deviation; SOL, sleep onset latency; TST, total sleep time; WASO, wake after sleep onset.

Table 6 summarizes the results under all four conditions. The WASO and arousal indices were the lowest in the PSG_alc_ and CPAP_alc_ groups_,_ respectively. Furthermore, N3 and REM durations were the highest in the CPAP_alc_ and CPAP_ctr_ groups, respectively. Respiratory indices, including AHI, were the lowest in the CPAP_ctr_ group.

**Table 6:** Summary of results.

Patient characteristic	PSG_ctr_	PSG_alc_	CPAP_ctr_	CPAP_alc_
	Mean ± SD	Mean ± SD	Mean ± SD	Mean ± SD
TST, min	369.98 ± 45.40	372.93 ± 32.89	376.10 ± 29.75	371.67 ± 31.67
Sleep efficiency, %	87.97 ± 10.81	88.91 ± 7.88	89.60 ± 7.09	88.65 ± 7.34
N1, %	19.93 ± 12.48	20.43 ± 12.47	10.64 ± 4.21	10.78 ± 4.06
N2, %	46.47 ± 13.38	47.61 ± 10.55	51.11 ± 8.40	51.95 ± 8.84
N3, %	4.62 ± 5.23	6.29 ± 6.23	6.52 ± 5.63	8.55 ± 6.43
REM, %	18.61 ± 6.40	16.85 ± 6.78	21.97 ± 6.01	18.93 ± 5.81
W, %	10.41 ± 10.47	8.83 ± 6.27	9.76 ± 6.28	9.80 ± 6.29
WASO, min	42.66 ± 41.40	34.89 ± 22.42	41.08 ± 26.58	41.02 ± 24.17
SOL, min	9.63 ± 16.47	11.10 ± 16.58	6.86 ± 12.28	6.59 ± 9.06
REM latency, min	99.19 ± 54.62	125.03 ± 54.78	81.91 ± 33.96	113.76 ± 53.26
AHI, /h	31.39 ± 28.17	32.69 ± 27.95	4.60 ± 4.59	5.37 ± 4.92
Supine AHI, /h	41.21 ± 35.87	41.22 ± 32.64	5.13 ± 5.40	5.95 ± 5.54
RDI, /h	37.49 ± 26.51	39.11 ± 25.75	7.10 ± 6.13	7.27 ± 5.14
Snoring rate, %	37.56 ± 20.20	45.47 ± 18.21		
Arousal index, /h	34.56 ± 22.19	33.93 ± 20.50	17.04 ± 7.05	15.79 ± 5.86
Mean oxygen saturation, %	94.77 ± 2.44	93.29 ± 3.46	96.52 ± 0.91	95.88 ± 1.22
Lowest oxygen saturation, %	82.23 ± 8.81	77.45 ± 10.63	89.17 ± 5.04	86.47 ± 7.00
T 90	7.47 ± 13.83	13.82 ± 19.41	0.26 ± 1.09	0.84 ± 2.19

All measurements are reported as means ± standard deviations. AHI, apnea–hypopnea index; PSG_ctr_, polysomnography without alcohol consumption; PSG_alc_, polysomnography after alcohol consumption; CPAP_ctr_, continuous positive airway pressure titration without alcohol consumption; CPAP_alc_, continuous positive airway pressure titration after alcohol consumption; N1, non-rapid eye movement sleep, stage 1; N2, non-rapid eye movement sleep, stage 2; N3, non-rapid eye movement sleep, stage 3; REM, rapid eye movement; RDI, respiratory disturbance index; SD, standard deviation; SOL, sleep onset latency; TST, total sleep time; WASO, wake after sleep onset; T90, percentage of total sleep time with oxygen saturation below 90%.

## Discussion

### Effects of CPAP on alcohol metabolism

After alcohol consumption, the ethanol and acetaldehyde levels varied. The levels measured in the morning could have shown variations depending on the use of CPAP and sleep status. Surprisingly, we also observed differences in the values measured before bedtime after consuming alcohol. All participants consumed an equal amount of alcohol under both conditions, and these measurements were not affected by CPAP use or sleep status as they were obtained before bedtime. Therefore, this phenomenon is likely due to the sensitivity of alcohol metabolism to variations in individual physical conditions, leading to metabolic differences even when the same amount of alcohol is consumed under identical conditions. From our findings, even when the same amount of alcohol was consumed under the same conditions, it was metabolized differently, leading to different levels of intoxication or hangover symptoms.

Our key findings indicate CPAP reduces acetaldehyde level. While CPAP further decreased BrAC, BAL, and AcAld by 4.2%, 4.7%, and 21.2%, respectively, only the reduction in AcAld reached statistical significance. Ethanol and acetaldehyde metabolism required oxygen; however, acetaldehyde metabolism appeared to be more oxygen-dependent. Oxygen plays an important role in alcohol metabolism^7^, which is likely to be inhibited in cases of hypoxia. Alcohol consumption has been reported to increase oxygen consumption to the point of liver hypoxia^8–10^. However, research on the impact of oxygenation status on alcohol metabolism is limited, with reports suggesting that oxygen-enriched alcoholic beverages may lead to a faster sobering process^11^. Similarly, alcohol metabolism can also be disrupted during intermittent hypoxia, and CPAP administration may, therefore, counteract this effect.

Alcohol metabolism occurs through three main pathways^7^. First, approximately 80% of alcohol is metabolized in the cytosol by alcohol dehydrogenase (ADH), which converts it into acetaldehyde. Second, approximately 10% of absorbed alcohol is broken down into acetaldehyde by CYP2E1 located in the microsomes. Finally, a small portion is metabolized by an enzyme known as catalase. The acetaldehyde generated is in turn metabolized into acetate by aldehyde dehydrogenase (ALDH). Both ADH and ALDH, which are involved in the metabolism of most of the alcohol consumed, require oxygen as an essential component^7^. ADH and ALDH reduce nicotinamide adenine dinucleotide (NAD+) into NADH. An adequate amount of oxygen is essential for NADH to be continuously oxidized to NAD^+^^7^. Moreover, CYP2E1 also requires oxygen for ethanol metabolism. In contrast, oxidative pathways mediated by catalase and non-oxidative mechanisms can operate without oxygen requirements; however, these pathways play minor roles. Additionally, alternative pathways could be activated as compensatory mechanisms when the main pathway is inhibited.

**Fig. 2:**
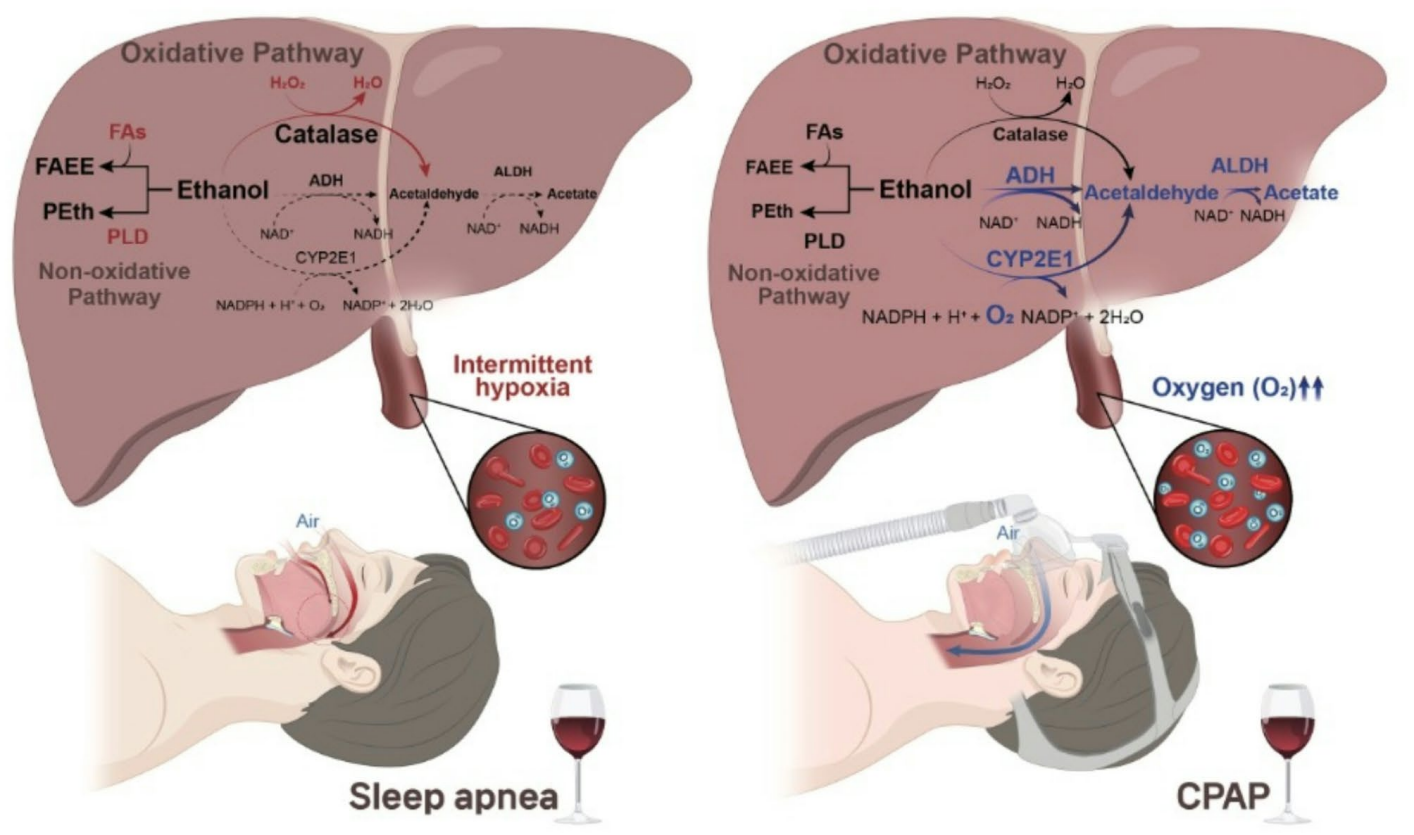
Summary of the effects of CPAP use on alcohol metabolism. FAEE, fatty acid ethyl ester; FAs, fatty acid ethyl ester synthase; Peth, Phosphatidyl ethanol; PLD, phospholipase D; ADH, alcohol dehydrogenase; ALDH, aldehyde dehydrogenase; NAD, Nicotinamide adenine dinucleotide; NADH, nicotinamide adenine dinucleotide dehydrogenase; NADPH, nicotinamide adenine dinucleotide phosphate dehydrogenase; NADP, nicotinamide adenine dinucleotide phosphate.

Previous studies have reported increased catalase activity during chronic alcohol administration or oxidative stress, including increased hydrogen peroxide levels^12,13^. Given that intermittent hypoxia can increase hydrogen peroxide levels^14–16^, this may also increase catalase activity, thereby promoting ethanol metabolism. Finally, when the oxidative pathway of ethanol is inhibited, metabolism occurs through non-oxidative pathways^17^. However, acetaldehyde is solely metabolized by ALDH; therefore, hypoxia severely inhibits its breakdown. Reportedly, hypoxia may induce liver damage in patients with sleep apnea^18^. To determine whether the results of our study were due to impaired liver function, we measured AST, ALT, and r-GT levels. However, no significant differences in the levels of these compounds were observed between the groups. Considering all these findings, we suggest that alcohol metabolism is inhibited in patients with snoring or sleep apnea. Although ethanol metabolism can still take place through alternative pathways that do not require oxygen, acetaldehyde metabolism is particularly inhibited because acetaldehyde is metabolized solely by ALDH, without available alternative pathways. However, CPAP administration can help restore normal metabolism after alcohol consumption (Fig. 2).

### Effects of alcohol consumption and CPAP on sleep quality

Our study revealed that alcohol consumption increased N3 and did not significantly worsen sleep apnea; however, it increased the snoring rate, reduced oxygen saturation, and suppressed REM. These findings are consistent with the existing literature^5,6^ and suggest that consuming alcohol ultimately reduces sleep quality, except for N3. Although alcohol did not significantly increase overall AHI or REM AHI, the increase in REM AHI (3.01 vs. 1.30) was slightly larger. Previous studies have reported a statistically significant increase in AHI with alcohol consumption, typically around 3.98^6^. While our results did not reach statistical significance, the observed AHI change was within a similar range as in prior studies, indicating no substantial difference. Furthermore, when CPAP was provided, sleep apnea improved to a normal level and the durations of N2, N3 and REM were increased, whereas that of N1 decreased, as did the arousal index. These findings indicate that CPAP is an effective treatment for sleep apnea and enhances sleep quality.

When the results of the CPAP_alc_ group were compared with those of the PSG_ctr_ group, we observed improved sleep quality, indicated by decreases in the AHI and arousal index and an increase in the N2 and N3 duration. This implies that CPAP administration after alcohol consumption improved sleep quality, compared with the sleep quality in individuals who did not consume alcohol. The most notable difference was observed in the REM when the CPAP_alc_ and PSG_ctr_ groups, and the CPAP_alc_ and PSG_alc_ groups were compared. As neither the PSG_ctr_ group nor the PSG_alc_ group used CPAP and differed only in terms of alcohol consumption, the differences observed when comparing each to the CPAP_alc_ group were due to differences in the separate and combined effects of CPAP and alcohol consumption. The CPAP_alc_ group showed higher REM duration than did the PSG_alc_ but similar REM duration compared with the PSG_ctr_ group. This can be attributed to the fact that CPAP alone increases REM, whereas alcohol suppresses it. However, when used simultaneously, compared with sleeping without CPAP or consuming alcohol, these two factors offset each other, resulting in no significant difference in REM. Additionally, in comparison to the PSG_ctr_ group, N3 exhibited a comparable increase in the PSG_alc_ and CPAP_ctr_ groups, respectively. However, the CPAP_alc_ group demonstrated the most pronounced elevation in N3. We believe that the combined effects of alcohol consumption and simultaneous CPAP may have a synergistic effect on this variable.

Using CPAP, we compared two groups based on whether they consumed alcohol or not. Significant differences were found between the two groups. The mean and lowest levels of oxygen saturation were higher in the non-alcohol consumption group than in alcohol consumption group. Additionally, the non-alcohol consumption group showed higher REM duration and lower REM latency than did their counterpart. This suggests that using CPAP without alcohol consumption may result in better sleep quality, particularly regarding oxygen saturation and REM sleep, compared with using CPAP after alcohol consumption. REM sleep has been reported to be particularly affected by alcohol consumption^5^, and we found that CPAP did not mitigate its negative effects on REM sleep. However, N3 duration was increased when CPAP was administered after alcohol consumption. Identifying the stage that promotes better sleep quality can be ambiguous, because it may vary based on individual sleep conditions.

Considering these findings together, we can infer that PSG_alc_ is the least helpful among the four conditions for sleep quality, followed by PSG_ctr_ and CPAP_alc._ The CPAP_ctr_ is most closely associated with good quality sleep, because it improves sleep apnea, increases oxygen saturation, N2, N3 and REM sleep, and reduces arousal. However, CPAP_alc_ is most effective at increasing N3 sleep.

Alcohol can lead to serious conditions, such as hypertension, arrythmia, stroke, fatty liver, and cirrhosis. Alcoholic beverages also contain many carcinogens, such as acetaldehyde, aflatoxin, arsenic, benzene, cadmium, and ethanol^1,2,4,5,19^. Among these compounds, acetaldehyde serves as a potent carcinogen and is classified as group 1 carcinogens by the International Agency for Research on Cancer^19^. Additionally, it can cause hangovers and lead to cardiovascular and hepatic diseases^20,21^. Therefore, as demonstrated by this study, considering the use of CPAP to reduce acetaldehyde levels in the body and preventing the harmful effects of alcohol consumption is important. Unfortunately, CPAP users are often less likely to use the CPAP device after consuming alcohol because they find it cumbersome and uncomfortable^22^. This subpopulation is even more in need of active treatment for sleep apnea or snoring to reduce hangovers and prevent various alcohol-related diseases, including cancer. To counteract the negative effects of alcohol consumption, several drugs have been developed to enhance ethanol metabolism and relieve hangovers^23,24^. Notably, the efficacy of these drugs has not been fully validated.

Our study is the first to confirm that CPAP can increase the breakdown of acetaldehyde as an alcohol metabolite, and improve sleep quality in alcohol users who snore or have sleep apnea.

This study has some limitations. First, the study was based on patients with complaints of snoring or sleep apnea, and the results may be different for other patient subpopulations. Second, this study was conducted in a sleep laboratory under controlled conditions, with a fixed quantity of alcohol consumed at a consistent rate. Consequently, the outcomes may not be generalizable to individuals who consume alcohol daily.

## Conclusions

Unlike previous studies that have relied on drugs or dietary components, we adopted an innovative approach to test methods aimed at improving alcohol metabolism by oxygen supplementation in individuals affected by snoring or sleep apnea. Our findings strongly recommend that these individuals use CPAP consistently after alcohol consumption to prevent its adverse effects.

## Methods

### Participants

Male participants aged ≥ 19 years who visited Ajou University Hospital (Suwon, Republic of Korea) for sleep disorders between January 2016 and July 2021, underwent PSG and CPAP titration, and consented to participate in this non-randomized crossover trial when asked at their outpatient appointment were recruited for this study. Among them, participants who regularly consumed an average of ≥ 1.0 g of alcohol per kilogram of bodyweight and were confirmed free of serious diseases were included. Individuals with liver diseases, malignancies, severe cardiovascular diseases, conditions that could affect alcohol metabolism, sleep-related hypoventilation, or Asian flush were excluded. Additionally, all subjects had not been diagnosed with a sleep-related breathing disorder or used CPAP prior to the study.

**Fig. 3:**
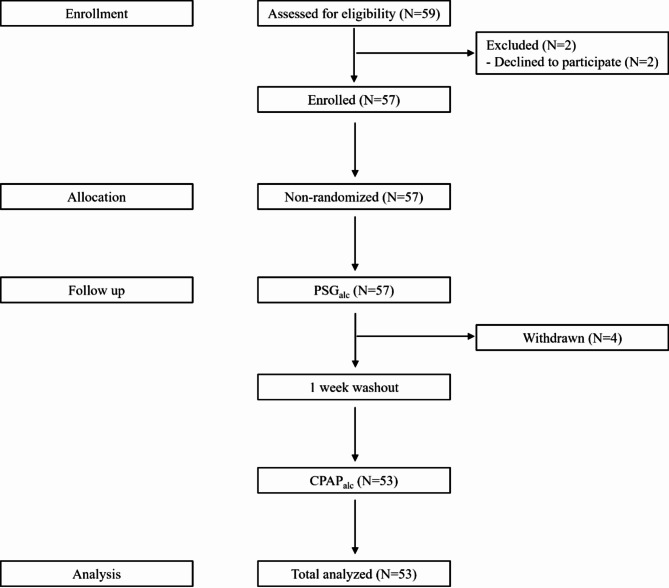
Flow diagram of participants included in the analysis. PSG_alc_, polysomnography after alcohol consumption; PSG_ctr_, polysomnography without alcohol consumption.

A total of 59 participants were recruited: two were excluded, resulting in 57 enrolled participants. During the study, four participants withdrew their participation for personal reasons; finally, 53 participants completed the study (Fig. 3). All statistical analyses and comparisons were conducted using data from the 53 participants who completed all four conditions.

### PSG and CPAP titration

PSG was conducted using an Embla N7000 device (Natus Medical, Middleton, WI). All PSG and CPAP measurements were performed and interpreted by a sleep expert according to the American Academy of Sleep Medicine recommendations^25,26^. In this study, all sleep parameters, including AHI, were obtained from polysomnography (PSG) rather than CPAP device recordings.

### Experimental procedures

A sleep technologist conducted the study. All participants underwent four different sleep tests, each involving both PSG and CPAP titration, conducted either with or without alcohol administration in the Ajou University Hospital Sleep Laboratory. The four specific tests conducted included (i) PSG without alcohol (PSG_ctr_), (ii) PSG with alcohol (PSG_alc_), (iii) CPAP titration without alcohol (CPAP_ctr_), and (iv) CPAP titration with alcohol (CPAP_alc_). Split-night polysomnography was not performed; instead, each session consisted of either a full-night polysomnography or a full-night CPAP titration. In sessions involving alcohol consumption, participants were provided with a high dose of Chamisul, a traditional Korean liquor, at a rate of 1.0 g per kilogram of bodyweight; Cham crackers (18.6 g, 84 cal); and water. On the study day, participants arrived at the sleep laboratory at 8 PM, drank alcohol from 9 to 10 PM at regular intervals to ensure consistent ingestion, and then brushed their teeth. At 10:30 PM, breath alcohol tests were performed, and blood samples were collected. The following morning, another set of breath alcohol tests and blood sampling was conducted.

### Measurement of blood alcohol level and breath alcohol concentration

BrAC was measured using a Lion Alcolmeter 500 instrument (Lion Laboratories Limited, Barry, UK), whereas BAL was determined using a cobas c503 analyzer (Roche, Basel, Switzerland).

### Measurement of the concentration of acetaldehyde in the blood

AcAld was analyzed using an acetaldehyde assay kit (Megazyme, Bray, Ireland).

### Liver function tests

The concentrations of AST, ALT, and r-GT were measured using the enzymatic rate method, with the Roche cobas c702 analyzer.

### Statistical analyses

The demographic characteristics of the study participants are presented as mean ± standard deviation (*SD*). Before conducting statistical comparisons, normality was assessed. For normally distributed data, paired *t*-tests were used to compare sleep study outcomes and alcohol metabolism-related measures between nighttime and morning sessions during PSG and CPAP conditions. If normality was not satisfied, the Wilcoxon signed-rank test was applied as a non-parametric alternative. Additionally, paired *t*-tests were conducted to compare sleep quality assessments between conditions with and without CPAP use and alcohol consumption. Agreement between measurements obtained from PSG and CPAP titration after alcohol intake was analyzed using Lin’s concordance correlation coefficient (CCC), where a CCC value of < 0.9 indicates poor to perfect agreement. To control for multiple comparisons, we applied the Bonferroni correction to ensure statistical validity. All statistical analyses were performed using R software, version 4.0.5 (R Foundation, Vienna, Austria).

### Study approval

The trial protocol and consent form were approved by the Ajou University Hospital Institutional Review Board (number: DEV-DE3-14-401). Written informed consent was obtained from all participants prior to any procedures. The trial was registered in the Clinical Research Information Service (CRIS) (https://cris.nih.go.kr; KCT0008792).

All methods were performed in accordance with the relevant guidelines and regulations.

## Electronic supplementary material

Below is the link to the electronic supplementary material.

Supplementary Material 1

## Data Availability

Individual, de-identified participant data will be shared by the corresponding author, Hyun Jun Kim (entkhj@ajou.ac.kr), upon reasonable request.
