# Supplementary material for: Enhanced alcohol metabolism and sleep quality with continuous positive airway pressure following alcohol consumption
Source: Sci Rep. 2025 Apr 28;15:14839. doi: 10.1038/s41598-025-98702-9 (PMC12037736; doi:10.1038/s41598-025-98702-9)
Supplement: Supplementary file 1 — Supplementary Material 1 [file 41598_2025_98702_MOESM1_ESM.docx]

Supplementary Information

Enhanced alcohol metabolism in liver and sleep quality with continuous positive airway pressure following alcohol consumption

Hyun Jun Kim, Do-Yang Park, Wee Gyo Lee, Kang Il Lee, Jin Ji Jung, Han Sang Lee, Sang In Hwang, Ji Hyun Park, Bumhee Park

Supplementary Table 1: Differences between PSG_alc_ and CPAP_alc_ measurements at night and in the morning

|  |  | PSG_alc_ - CPAP_alc_ | | | PSG_alc_ - CPAP_alc_ /  PSG_alc_ (%) |
| --- | --- | --- | --- | --- | --- |
|  |  | Mean±SD | CCC | 95% CI |  |
| Night | BrAC | 0.03± 0.02 (g%) | 0.31 | 0.07 to 0.52 | 32.67±26.04 |
|  | BAL | 31.65±26.31 (mg/dL) | 0.26 | 0.00 to 0.48 | 34.27±36.49 |
|  | AcAld | 1.45±1.54 (mg/L) | 0.54 | 0.32 to 0.70 | 29.16±30.6 |
| Morning | BrAC | 0.01±0.01 (g%) | 0.29 | 0.08 to 0.49 | NA |
|  | BAL | 10.83±12.75 (mg/dL) | 0.42 | 0.19 to 0.61 | NA |
|  | AcAld | 1.32±0.84 (mg/L) | 0.35 | 0.16 to 0.51 | 71.25±132.52 |

All measurements are reported as means±standard deviations.

Abbreviations: BrAC, breath alcohol concentration; BAL, blood alcohol level; AcAld, acetaldehyde concentration in the blood; CCC, Lin’s concordance correlation coefficient; CI, confidence interval; NA, not applicable (in cases where certain parameters had a null value, relative changes could not be estimated in the morning BrAC and BAL, and thus, they appear as NA); PSG_alc_, polysomnography after alcohol consumption; CPAP_alc_, continuous positive airway pressure titration after alcohol consumption

Supplementary Table 2. Results of liver function test

|  |  | PSG_alc_ | CPAP_alc_ | *p* value |
| --- | --- | --- | --- | --- |
| Differences  between  night and  morning | AST (U/L) | -0.79±22.86 | -1.06±19.16 | 0.926 |
|  | ALT (U/L) | -1.91±19.06 | -2.41±9.81 | 0.850 |
|  | r-GT (U/L) | 2.87±8.49 | 1.69±5.85 | 0.368 |

All measurements are reported as means±standard deviations.

AST, aspartate transaminase; ALT, alanine aminotransferase; r-GT, r-glutamyl transferase; PSG_alc_, polysomnography after alcohol consumption; CPAP_alc_, continuous positive airway pressure titration after alcohol consumption.

Changes and paired t-test of AST, ALT, and r-GT values measured at night and in the morning during PSG and CPAP sessions.

Supplementary Table 3: Changes in sleep status according to alcohol consumption

|  | PSG_ctr_ | PSG_alc_ | PSG_ctr_ - PSG_alc_ | 95% confidence interval | | *p* value |
| --- | --- | --- | --- | --- | --- | --- |
| Patient characteristic | Mean±SD | Mean±SD | Mean±SD | L | U |  |
| TST, min | 369.98±45.40 | 372.93±32.89 | -2.95±46.12 | -15.66 | 9.76 | 0.643 |
| Sleep efficiency, % | 87.97±10.81 | 88.91±7.88 | -0.94±11.14 | -4.01 | 2.13 | 0.543 |
| N1, % | 19.93±12.48 | 20.43±12.47 | -0.50±8.08 | -2.72 | 1.73 | 0.657 |
| N2, % | 46.47±13.38 | 47.61±10.55 | -1.13±12.35 | -4.54 | 2.27 | 0.507 |
| N3, % | 4.62±5.23 | 6.29±6.23 | -1.67±5.22 | -3.11 | -0.23. | 0.024* |
| REM, % | 18.61±6.40 | 16.85±6.78 | 1.76±6.31 | 0.03 | 3.50 | 0.047* |
| W, % | 10.41±10.47 | 8.83±6.27 | 1.58±10.94 | -1.43 | 4.60 | 0.298 |
| WASO, min | 42.66±41.40 | 34.89±22.42 | 7.77±43.44 | -4.20 | 19.74 | 0.199 |
| SOL, min | 9.63±16.47 | 11.10±16.58 | -1.47±14.90 | -5.57 | 2.64 | 0.477 |
| REM latency, min | 99.19±54.62 | 125.03±54.78 | -25.84±64.01 | -43.48 | -8.20 | 0.005** |
| AHI, /h | 31.39±28.17 | 32.69±27.95 | -1.30±11.75 | -4.54 | 1.94 | 0.423 |
| Supine AHI, /h | 41.21±35.87 | 41.22±32.64 | 0.01±9.94 | -2.75 | 2.73 | 0.997 |
| REM AHI | 34.59±24.48 | 37.61±24.83 | -3.01±17.84 | -7.93 | 1.90 | 0.224 |
| RDI, /h | 37.49±26.51 | 39.11±25.75 | -1.62±11.88 | -4.89 | 1.66 | 0.326 |
| Snoring rate, % | 37.56±20.20 | 45.47±18.21 | -7.91±18.22 | -12.93 | -2.89 | 0.003** |
| Arousal index, /h | 34.56±22.19 | 33.93±20.50 | 0.63±13.05 | -2.97 | 4.22 | 0.727 |
| Mean oxygen saturation, % | 94.77±2.44 | 93.29±3.46 | 1.49±1.59 | 1.05 | 1.93 | 0.000*** |
| Lowest oxygen saturation, % | 82.23±8.81 | 77.45±10.63 | 4.77±5.11 | 3.37 | 6.18 | 0.000*** |

All measurements are reported as means±standard deviations. * *p* < 0.05, ** *p* < 0.01, *** *p* < 0.001

Abbreviations: AHI, apnea–hypopnea index; N1, non-rapid eye movement sleep, stage 1; N2, non-rapid eye movement sleep, stage 2; N3, non-rapid eye movement sleep; PSG_alc_, polysomnography after alcohol consumption; PSG_ctr_, polysomnography without alcohol consumption; REM, rapid eye movement; RDI, respiratory disturbance index; SD, standard deviation; SOL, sleep onset latency; TST, total sleep time; WASO, wake after sleep onset

Supplementary Table 4: Changes in sleep status according to the use of CPAP

|  | PSG_ctr_ | CPAP_ctr_ | PSG_ctr_ - CPAP_ctr_ | 95% confidence interval | | *p* value |
| --- | --- | --- | --- | --- | --- | --- |
| Patient characteristics | Mean±SD | Mean±SD | Mean±SD | L | U |  |
| TST, min | 369.98±45.40 | 376.10±29.75 | -6.12±53.37 | -20.83 | 8.59 | 0.408 |
| Sleep efficiency, % | 87.97±10.81 | 89.60±7.09 | -1.63±12.75 | -5.14 | 1.89 | 0.357 |
| N1, % | 19.93±12.48 | 10.64±4.21 | 9.29±13.31 | 5.62 | 12.96 | 0.000*** |
| N2, % | 46.47±13.38 | 51.11±8.40 | -4.64±12.70 | -8.14 | -1.14 | 0.010** |
| N3, % | 4.62±5.23 | 6.52±5.63 | -1.90±6.24 | -3.62 | -0.18 | 0.031 |
| REM, % | 18.61±6.40 | 21.97±6.01 | -3.35±8.88 | -5.80 | -0.91 | 0.008* |
| W,% | 10.41±10.47 | 9.76±6.28 | 0.65±11.73 | -2.58 | 3.89 | 0.687 |
| WASO, min | 42.66±41.40 | 41.08±26.58 | 1.58±46.39 | -11.21 | 14.37 | 0.805 |
| SOL, min | 9.63±16.47 | 6.86±12.28 | 2.77±11.05 | -0.28 | 5.82 | 0.074 |
| REM latency, min | 99.19±54.62 | 81.91±33.96 | 17.28±61.35 | 0.37 | 34.19 | 0.045* |
| AHI, /h | 31.39±28.17 | 4.60±4.59 | 26.78±25.87 | 19.65 | 33.91 | 0.000*** |
| Supine AHI, /h | 41.21±35.87 | 5.13±5.40 | 36.08±33.90 | 26.74 | 45.43 | 0.000*** |
| RDI, /h | 37.49±26.51 | 7.10±6.13 | 30.39±23.81 | 23.83 | 36.96 | 0.000*** |
| Arousal index, /h | 34.56±22.19 | 17.04±7.05 | 17.52±22.66 | 11.27 | 23.76 | 0.000*** |
| Mean oxygen saturation, % | 94.77±2.44 | 96.52±0.91 | -1.74±2.30 | -2.38 | -1.11 | 0.000*** |
| Lowest oxygen saturation, % | 82.23±8.81 | 89.17±5.04 | -6.94±6.47 | -8.73 | -5.16 | 0.000*** |

All measurements are reported as means±standard deviations. * *p* < 0.05, ** *p* < 0.01, *** *p* < 0.001

Abbreviations: AHI, apnea–hypopnea index; CPAP_ctr_, continuous positive airway pressure titration without alcohol consumption; N1, non-rapid eye movement sleep, stage 1; N2, non-rapid eye movement sleep, stage 2; N3, non-rapid eye movement sleep; PSG_ctr_, polysomnography without alcohol consumption; REM, rapid eye movement; RDI, respiratory disturbance index; SD, standard deviation; SOL, sleep onset latency; TST, total sleep time; WASO, wake after sleep onset.
